# Targeted electroporation of defined lateral ventricular walls: a novel and rapid method to study fate specification during postnatal forebrain neurogenesis

**DOI:** 10.1186/1749-8104-6-13

**Published:** 2011-04-05

**Authors:** María E Fernández, Simona Croce, Camille Boutin, Harold Cremer, Olivier Raineteau

**Affiliations:** 1 Brain Research Institute, University of Zürich/ETHZ, CH-8057 Zürich, Switzerland; 2 IBDML,13288 Marseille, France

## Abstract

**Background:**

Postnatal olfactory bulb (OB) neurogenesis involves the generation of granule and periglomerular cells by neural stem cells (NSCs) located in the walls of the lateral ventricle (LV). Recent studies show that NSCs located in different regions of the LV give rise to different types of OB neurons. However, the molecular mechanisms governing neuronal specification remain largely unknown and new methods to approach these questions are needed.

**Results:**

In this study, we refine electroporation of the postnatal forebrain as a technique to perform precise and accurate delivery of transgenes to NSCs located in distinct walls of the LV in the mouse. Using this method, we confirm and expand previous studies showing that NSCs in distinct walls of the LV produce neurons that invade different layers of the OB. Fate mapping of the progeny of radial glial cells located in these distinct LV walls reveals their specification into defined subtypes of granule and periglomerular neurons.

**Conclusions:**

Our results provide a baseline with which future studies aiming at investigating the role of factors in postnatal forebrain neuronal specification can be compared. Targeted electroporation of defined LV NSC populations will prove valuable to study the genetic factors involved in forebrain neuronal specification.

## Background

Because of its persisting neurogenesis throughout life, the olfactory bulb (OB) is a region of particular interest to study the development and continuous remodeling of neuronal circuits.

Granule cells and periglomerular (PG) cells are the two main classes of OB inhibitory interneurons that continue to be generated after birth from stem cells located in the subventricular zone (SVZ). The peak of granule cell production occurs during the first postnatal week [1]. PG interneurons can be divided into different subtypes based on their expression of tyrosine hydroxylase (TH) or of the calcium binding proteins calretinin (CR) or calbindin (CB) [2]. These populations of PG interneurons have different temporal origins, with birth of dopaminergic (TH+) neurons predominating during embryonic development, and birth of interneurons expressing CR predominating in adult stages [3]. At early postnatal stages, however, the three subtypes of PG interneurons are produced in equal quantities [3].

Recent studies have revealed continuity in the development of forebrain germinal zones, with many regions of the embryonic neuroepithelium (that is, the medial and lateral ganglionic eminence as well as the pallium) contributing to the adult SVZ [4]. In agreement with their distinct developmental origin, the lateral ventricle (LV) walls contain progenitor populations that are biased to acquire defined OB neuronal fates [4-6]. This early specification appears to reflect an intrinsic property of the cells, as defined by ectopic transplantation experiments [5,6].

Together, these observations raise important questions on the plastic potential of SVZ progenitors as well as on the transcriptional regulation of neuronal specification in the postnatal OB. Unraveling the transcriptional cues acting in the specification of OB interneurons necessitates the establishment of new techniques that would allow rapid and accurate manipulation of single or multiple genes in defined regions of the LV. Electroporation has recently appeared as an efficient means to deliver transgenes into resident NSCs of the postnatal and, to a lesser extent, of the adult forebrain [7-9]. This technique has rapidly become the method of choice to manipulate gene expression in NSCs of the postnatal forebrain, permitting the study of factors influencing the maintenance of SVZ neural stem cell (NSC) pools [10], the control of newborn neuron differentiation [11], and the survival of migrating neuroblasts [12].

Here we refine and improve the electroporation technique by showing that it can be accurately and reproducibly used to target transgene delivery to specific walls of the LV. Furthermore, we fate map the progeny of radial glial cells (RGC); the perinatal neural stem cells) located in distinct walls of the postnatal LV, providing a baseline with which future studies aiming at investigating the role of factors in neuronal specification can be compared.

## Results

### Spatial accuracy of electroporation using electrodes of different diameters

**Figure 1:**
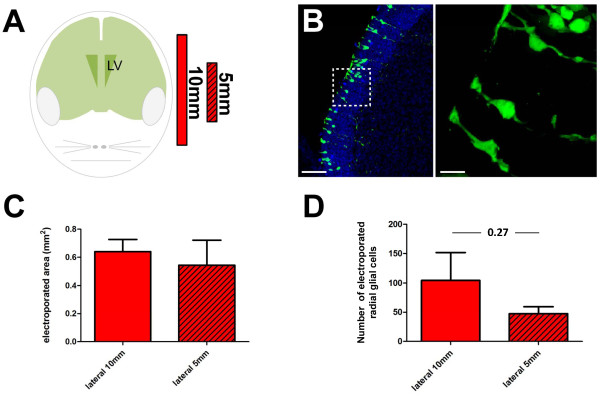
**Electroporation efficiency using electrodes of different sizes**. **(A) **Schematic representation of the position and size (10 mm and 5 mm) of the electrodes used in the first part of this study. Only the positive pole is represented. **(B) **Representative example of electroporated radial glial cells (RGCs) expressing high levels of GFP at 1 day post-electroporation. The right panel shows a higher magnification of the region surrounded by the dotted box. Only cells with clear RGC morphology, that is, with an end foot contacting the ventricle surface and a main apical process, were counted. DAPI (blue) was used as a nuclear counterstain. **(C,D) **Measurements of the electroporated area (C) and of the number of electroporated RGCs (D) when 10-mm (filled bar) or 5-mm (hatched bars) diameter electrodes were used. Please refer to Materials and methods and Additional file 1 for experimental details. Note that the use of smaller electrodes does not improve the precision of electroporation (that is, did not decrease the electroporated area size), but tend to decrease the number of electroporated cells. Error bars represent standard error of the mean. Scale bars: 50 μm and 10 μm in (B) (left and right panels, respectively). LV, lateral ventricle.

We first compared the efficiency of transfection and the spatial distribution of electroporated cells using tweezer electrodes of 5 or 10 mm diameter placed on both sides of the animal head (Figure 1A).

**Figure 2:**
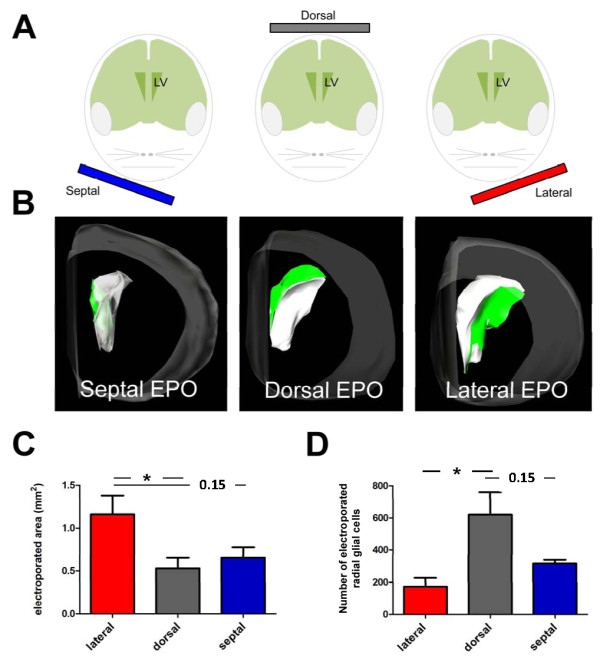
**Electroporation efficiency using different electrode locations**. **(A) **Schematic representation of the location of the electrodes used in the second part of this study. Only the positive pole is represented. Note the very ventral location of the tweezer-electrode cathode to target the lateral and septal wall of the lateral ventricle (LV), with no transfection of the dorsal wall. **(B) **Representative three-dimensional reconstruction illustrating the location and extent of the electroporated area after electroporation (EPO) with the electrode position shown in (A). Gray contours, brain; white contours, lateral ventricle; green contours, area containing electroporated cells. **(C,D) **Measurements of the electroporated area (C) and of the number of electroporated RGCs (D) obtained with the three electrode positions tested. Error bars represent standard error of the mean. *P < 0.05 as determined by unpaired *t*-test.

For this, we assessed the number of RGCs expressing GFP 1 day after the electroporation of a GFP-encoding plasmid (Figure 1B); throughout the rostro-caudal extent of the LV. For the analysis, one out of every three sections from the most rostral portion of the LV (that is, before the ventricle opens) up to the emergence of the dentate gyrus was considered, representing approximately 1.25 mm of tissue. On each section, the brain and ventricle outlines were drawn using the Neurolucida software (mbf Bioscience Williston, Vermont, USA Additional file 1). Next, the position of GFP+ RGCs (Figure 1B) was superimposed on the drawings, and a line defining the extension of the electroporated area was added (Additional file 1). A three-dimensional representation of the drawing was generated using Neurolucida explorer, allowing accurate measurement of the electroporated area and a three-dimensional reconstruction to be made (Figure 2B).

Using this approach, we found that lateral electroporation using a 10- or 5-mm diameter electrode resulted in comparable electroporated areas (Figure 1C; 0.64 ± 0.09 mm^2 ^and 0.55 ± 0.17 mm^2^, respectively, *P *= 0.64). However, the number of electroporated RGCs was approximately halved when 5-mm diameter tweezer electrodes were used, although this difference was not statistically significant (Figure 1D; 47 ± 12 versus 104.4 ± 47, n = 5 animals, *P *= 0.27).

Together, these initial observations indicate that the use of tweezer electrodes with a smaller diameter does not increase the spatial accuracy of electroporation of the LV. Furthermore, larger electrodes allow the transfection of a higher number of cells. The use of 10-mm electrodes was therefore preferred and used for all subsequent experiments.

### Efficient electroporation of the distinct LV walls

In light of recent studies showing the contribution of distinct LV walls to OB neurogenesis [4,5], we next tested if transgenes could be accurately delivered to defined walls of the LV by electroporation.

Tweezer electrodes were placed on both sides of pups' heads, and the positive electrode was differentially oriented to target the distinct walls of the LV (Figure 2A). The efficiency of transfection as well as the transfected area was measured 1 day post-electroporation (dpe), as explained above. All electrode positions resulted in efficient electroporation of RGCs in large areas of the LV (Figure 2B; Additional files 2, 3 and 4, which show rotating three-dimensional reconstructions of the electroporated area obtained with the three electrode positions). However, we could observe differences between the experimental groups regarding the respective sizes of the electroporated areas. Dorsal electroporation resulted in the smallest electroporated area (0.53 mm^2 ^± 0.12), followed by the septal (0.65 mm^2 ^± 0.12), and lateral electroporation (1.16 mm^2 ^± 0.22) (Figure 2C).

Analysis of the total number of GFP+ RGCs revealed that dorsal electroporation resulted in the largest number of electroporated RGCs (620 ± 140 GFP+ cells), while septal electroporation resulted in the electroporation of 316 ± 24 cells and lateral electroporation resulted in the smallest number of electroporated cells (173 ± 54 GFP+ cells) (Figure 2D).

**Figure 3:**
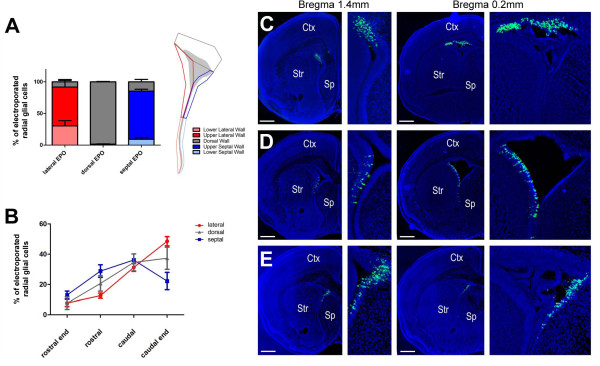
**Targeted electroporation of defined lateral ventricle wallS**. **(A) **Quantification of the percentage of GFP+ RGCs in subregions of the LV following lateral, dorsal and septal electroporation. Note the efficient targeting of distinct LV walls with different positioning of the electrodes. The most ventral regions of the lateral and septal LV walls, however, show lower numbers of electroporated cells. Error bars represent standard error of the mean. **(B) **Quantification of the percentage of GFP+ RGCs at defined rostro-caudal levels of the LV. **(C-E) **Representative overviews of the distribution of electroporated (GFP+, green) cells at two rostro-caudal levels of the LV (that is, Bregma 1.4 mm and 0.2 mm). DAPI (blue) was used as a nuclear counterstain. Scale bars: 1 mm. Ctx, cortex; EPO, electroporation; Sp, septum; Str, striatum.

To assess the accuracy of the targeted electroporation, we analyzed the dorso-ventral distribution of the electroporated cells by counting the number of GFP+ RGCs in five subregions, including the lateral, dorsal and septal walls of the LV (Figure 3A). We found that dorsal electroporation resulted in an almost exclusive targeting of cells in the dorsal region (98.5 ± 0.6% of the total amount of cells; Figure 3A,C). Lateral electroporation resulted in the targeting of the great majority of cells in the lateral wall (91.8 ± 3.8%; Figure 3A,D), and septal electroporation targeted 85.3 ± 5.6% of cells in the septal wall (Figure 3A,E). While the position of the electrodes to target the lateral and septal walls was very ventral, it is worth noting that a greater proportion of electroporated cells was found in the most dorsal regions of these walls (Figure 3A,C-E). Analysis of the rostro-caudal distribution of the GFP+ cells showed that dorsal and lateral electroporation resulted in a higher number of electroporated cells in the caudal regions of the periventricular zone, while septal electroporation resulted in GFP+ cells more evenly distributed throughout the rostro-caudal axis of the LV walls (Figure 3B-E).

Thus, reproducible specific targeting of each wall of the ventricle can be achieved with a minimal contamination of the other walls by varying the positioning of the electrodes around the head of the pup.

### Fate of electroporated cells located in defined lateral ventricle walls

RGCs generate committed neuroblasts that migrate to the OB where they locally differentiate into different subtypes of interneurons [13]. Two types of interneurons continue to be produced postnatally in the OB: PG cells located in the glomerular layer (GL), and granule neurons distributed throughout the mitral cell layer, internal plexiform layer and granule cell layer (GCL).

We used targeted electroporation to analyze the contribution of RGCs located in the different walls of the LV to the generation of diverse subpopulations of OB interneurons. First, we analyzed at 21 dpe the distribution of GFP+ cells in five defined layers in the OB (the peduncle, inner GCL, outer GCL, mitral cell layer/internal plexiform layer and GL).

**Figure 4:**
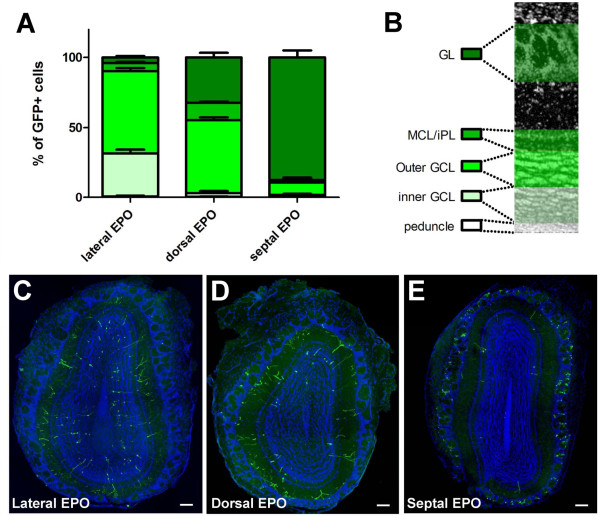
**Radial glial cells located in defined walls of the postnatal lateral ventricle produce neurons that migrate to distinct layers of the olfactory bulb**. **(A,B) **Quantification of the percentage of GFP+ newborn neurons in defined layers of the OB at 21 dpe. The OB layers are illustrated in (B). Error bars represent standard error of the mean. **(C-E) **Representative overviews of the distribution of newborn neurons in the OB of laterally, dorsally, and septally electroporated animals. DAPI (blue) was used as a nuclear counterstain. Scale bars: 100 μm. GCL, granule cell layer; EPO, electroporation; GL, glomerular layer; iPL, internal plexiform layer; MCL, mitral cell layer.

Results show clear changes in the distribution of the newly born neurons depending on their site of origin (Figure 4). Whereas RGCs located in the lateral wall generated mostly granule neurons (95.3 ± 1.3%), the septal wall generated almost exclusively PG neurons (88.8 ± 3.9%). Animals that were electroporated in the dorsal wall showed an intermediate picture, with 32.2 ± 3.4% of the GFP+ cells becoming PG neurons, while 67.5 ± 3.5% acquired a granule cell fate. Among the three groups of animals, only those electroporated in the lateral wall generated a significant population of deep granule cells, that is, cells in the inner GCL (30.92 ± 2.70% versus 2.90 ± 1.51% versus 0.58 ± 0.52% in the lateral, dorsal and septal wall electroporated groups, respectively; *P *< 0.001). Very few GFP+ cells were found in the peduncle (0.69 ± 0.41%, 0.38 ± 0.24% and 0.87 ± 0.78% arising from the lateral, dorsal and septal wall electroporated groups, respectively), indicating that most cells have reached their final location at this late time point.

These results establish that, at the perinatal stage, RGCs located in the dorsal wall of the LV give rise preferentially to neurons that populate the outer layers of the OB, while RGCs situated in the lateral wall produce neurons that remain in deeper layers of the OB. At this age, the septal wall almost exclusively gives rise to neurons in the GL.

**Figure 5:**
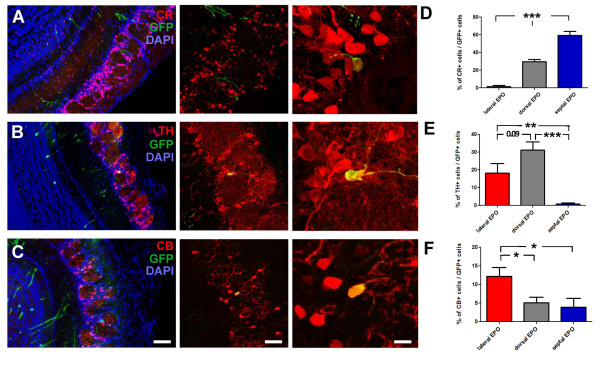
**Radial glial cells located in defined walls of the lateral ventricle specify distinct periglomerular neuron subtypes**. **(A-C) **Representative immunostainings for the three neuronal subtype markers CR (A), TH (B), and CB (C). For all markers, a low magnification overview with DAPI as a nuclear counterstain is shown (left panel), as well as confocal pictures showing higher magnifications of GFP+ neurons positive for the three markers (right panels). **(D-F) **Quantification of the percentage of newborn neurons (that is, GFP+) expressing CR (D), TH (E) or CB (F), which derived from the lateral (red), dorsal (gray) or septal (blue) LV walls. Error bars represent standard error of the mean. *P < 0.05; **P < 0.01; ***P < 0.001; determined by unpaired *t*-test. Scale bars: 50 μm, 30 μm and 10 μm in the left, middle and right panels, respectively. CB, calbindin; CR, calretinin; EPO, electroporation; TH, tyrosin hydroxylase.

Second, we studied the specification of the progeny of electroporated cells in the GL of the OB. Distinct non-overlapping populations of PG interneurons can be identified using the markers CR, TH and CB (Figure 5A-C). Again, clear differences in the subtypes of PG neurons generated by defined walls of the LV were observed (Figure 5D-E). Whereas only few GFP+ neurons generated by the lateral wall of the LV acquired a CR+ phenotype (1.3 ± 1.3%), this number increased for dorsal (29.5 ± 2.5%) and septal wall (59.1 ± 4.7%) electroporated animals (Figure 5D). A preferential dorsal origin was observed for TH+ neurons, with 31.3 ± 4.9% of GFP+ neurons originating from the dorsal wall of the LV acquiring this marker, while only 18.3 ± 4.5% and 0.9 ± 0.6% of GFP+ neurons originating in the dorsal and septal wall, respectively, expressed this marker (Figure 5E). Similarly, CB fate acquisition was mostly observed for neurons originating from the lateral wall (12.3 ± 2.4%), while only 5.1 ± 1.5% and 3.9 ± 2.3% of GFP+ neurons originating in the dorsal and septal wall, respectively, expressed this marker (Figure 5F). It should be noted, however, that the percentage of newborn neurons acquiring CB expression remained very low compared to those acquiring TH or CR expression.

**Figure 6:**
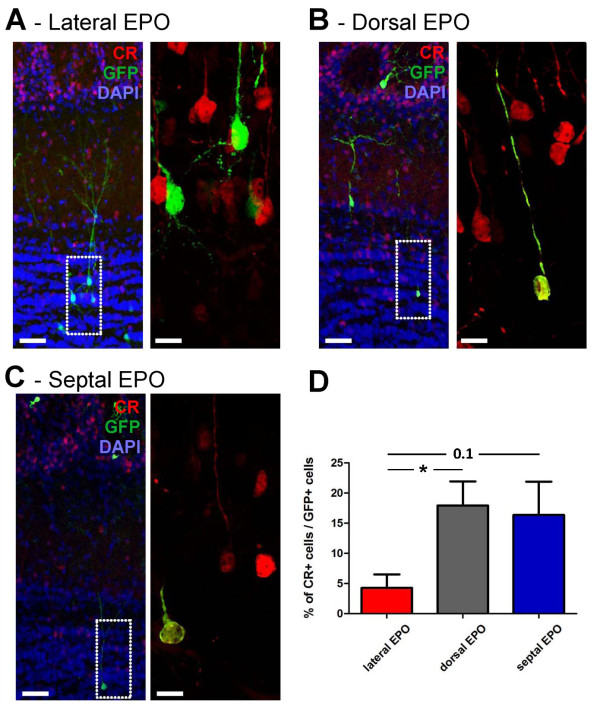
**Radial glial cells located in defined walls of the lateral ventricle produce distinct granule neuron subtypes**. **(A-C) **Representative immunostainings for CR expression in the GCL of animals electroporated in the lateral (A), dorsal (B) or septal (C) walls of the LV. For all groups, a low magnification overview (left panel) as well as a higher magnification of the underlined region (right panel) are presented. DAPI (blue) was used as a nuclear counterstain. **(D) **Quantification of the percentage of newborn granule cells (GFP+) expressing CR that derived from the lateral (red), dorsal (gray) or septal (blue) LV walls. Error bars represent standard error of the mean. *P < 0.05 as determined by unpaired *t*-test. Scale bars: 50 μm and 10 μm in the left and right panels, respectively. CR, calretinin; EPO, electroporation.

We next investigated if differential specification of granule CR+ neurons by defined LV walls paralleled that of PG neurons. The generation of granule cell subtypes by distinct LV walls was less clear than for PG neurons. Thus, whereas only 4.2 ± 2.2% of the granule cells generated from the lateral wall of the LV were CR+, larger numbers of CR+/GFP+ granule cells were generated from the dorsal and septal walls of the LV (17.9 ± 3.9% and 16.4 ± 5.5%, respectively; Figure 6).

Together, these results demonstrate a clear linkage between the regionalization of NSCs in the LV and the location and specification of their progeny in the OB. Thus, defined NSCs populations can be efficiently targeted by electroporation, allowing the genetic study of neuronal specification in the postnatal forebrain.

## Discussion

Over the past years, postnatal electroporation has become an important tool to manipulate gene expression in OB neural precursor cells. In this study we further refine this method by showing that plasmid delivery can be achieved with a high degree of accuracy and minimal variability in the lateral, dorsal and septal walls of the perinatal LV. Furthermore, we show that neurons produced from these distinct regions migrate to different OB layers and acquire defined subtype-specific markers. This approach therefore provides optimal readouts for rapidly exploring molecular cues, and in particular transcriptional factors involved in the establishment of LV organization as well as in the specification of different cell types in the postnatal forebrain.

Our results show that postnatal electroporation led to efficient plasmid delivery into RGCs independently of the area targeted. Decreasing the size of the electrodes resulted in less GFP+ RGCs with no gain in precision. Successfully transfected RGCs could be readily identified because of their unique morphological characteristics, that is, an end foot contacting the LV wall and an apical process extending to the brain surface. Previous studies have described in detail the phylogeny of RGCs and their involvement in the generation of astrocytes, oligodendrocytes and neurons at different developmental and postnatal stages [13-15]. At 21 dpe, RGCs from all LV walls of the LVs gave rise to numerous neurons in the OB. They differed, however, in their capacity to generate astrocytes and oligodendrocytes that remained in the vicinity of the corresponding LV walls, with mostly dorsal and septal wall RGCs giving rise to both cell populations (data not shown), in agreement with a previous study [14]. Thus, in addition to studying neuronal specification, targeted electroporation of the LV walls may allow the study of the molecular cues governing astrogenesis and oligodendrogenesis in the postnatal forebrain.

Targeted electroporation of distinct LV walls revealed clear differences in the spatial origin of granule cell subtypes. Granule cells of the OB can be classified by the position of their cell body in the GCL [16]. Those that have a cell body located in the innermost region of the GCL (that is, deep granule cells) present dendritic arborizations that ramify in the deep external plexiform layer, while those of superficial granule cells arborize in the most superficial part of the external plexiform layer, resulting in a preferential modulation of mitral and tufted cell activity, respectively [6,17]. Interestingly, these distinct granule cell subtypes show distinct rates of turnover after birth [18]. Our results show that, at postnatal day 2, most granule cells derive from the lateral and dorsal LV walls, with only a very small fraction of cells deriving from the septal wall. Moreover, we report a clear spatial relationship between the ventro-dorsal origin of granule cells in the LV and their migration to deep and superficial regions of the GCL. These results add to a previous study reporting that deep granule cells were produced by the posterior LV, while superficial granule cells were preferentially generated from the anterior LV [6]. Importantly, the rostro-caudal distribution of the GFP+ cells in the LV was comparable between the dorsally and laterally electroporated groups in our study, and cannot therefore account for our results.

In contrast to granule cells, the proportion of cells specifying PG neurons was higher for RGCs located in the dorsal and septal LV walls. The contribution of the most anterior region of the septal LV wall to PG neuron production has previously been reported [5]. The targeting of large caudal areas of the septal LV wall in our study, together with the large number of newborn neurons observed at 21 dpe in the OB, suggest that the contribution of this LV wall to early postnatal OB neurogenesis is larger than previously thought.

Subpopulations of PG interneurons are defined by their expression of the three non-overlapping markers CR, CB and TH [2,19]. In neonatal animals, fate mapping of progenitor cells expressing Dlx1/2-Cre have shown the generation of an equal proportion of PG neurons expressing these three markers [3]. In regard to these previous findings, the proportion of PG CB+ neurons labeled by electroporation (<10% of the newborn GL interneurons) might appear surprisingly low. This discrepancy might result from a low rate of transfection of the population of RGCs giving rise to this subtype of interneuron. Indeed, previous studies suggest that CB+ PG interneurons originate in the ventral most region of the LV [5], a region that shows a low rate of transfection as revealed by our three-dimensional reconstructions of the electroporated area. This low rate of transfection might be due to weaker diffusion of the plasmid to this narrow ventricular region. In agreement with previous studies [4,5], our results show that these three subtypes of PG interneurons largely originate from distinct LV walls. Thus, a greater proportion of RGCs from the lateral LV generate CB+ neurons, while those from the dorsal and septal wall preferentially generate TH+ and CR+ neurons, respectively. The very marked contribution of the septal wall to CR+ PG neurogenesis is likely to occur only at the perinatal age, as this LV region is not known to be neurogenic at later stages, while the birth of CR+ PG cells persists well into adulthood [3]. It is also interesting to note that whereas the majority of the PG neurons originating from the septal wall of the LV acquired a CR+ phenotype, only less than 20% of the few granule cells generated from the same region acquired expression of this marker. These results therefore suggest dissociation between the transcriptional regulation of CR+ phenotype acquisition by granule cells and PG cells.

Efficient genetic manipulation of NSCs in defined walls of the LV can be achieved by several approaches. Thus, the use of specific Cre mouse lines - Gsh2 and Emx1 - allow the targeting of NSCs located in the lateral and dorsal walls of the LV, respectively [4,20]. Transduction of discrete populations of RGCs by injection of an AAV-Cre to their apical processes also efficiently targets cells in defined walls of the LV [5,14]. We show here that targeted electroporation of the LV walls represents a viable alternative to these techniques, and offer a number of advantages. First, electroporation does not necessitate the breeding of particular mice strains or production of viral particles. Second, electroporation allows the concomitant manipulation of single or multiple genes, as previously demonstrated [8]. Finally, both shRNA or overexpression plasmids can be used [11], therefore offering opportunities for concomitant loss and gain-of-function studies. It should be mentioned, however, that this technique is unlikely to be suitable to study forebrain neurogenesis in the adult intact forebrain. At this late stage, electroporation only rarely targets neural stem cells (<1%) ([7] and our own observations), preventing the thoughtful quantification of defined lineages that might be targeted by electroporation.

## Conclusions

The vast possibilities that the approach presented here offers will prove to be of crucial help for unraveling the transcriptional codes acting in OB neuron specification in the postnatal forebrain. Many transcription factors - Dlx2, Pax6, Sp8, ER81, Neurog2, Tbr2, Gsh2, Emx1, and Mash1 - have been shown to be expressed in the walls of the LV with diverse degrees of spatial distribution [4,21-24]. Among these transcription factors, only the functions of Dlx2 and Pax6 in postnatal OB neuron specification have been thoroughly assessed [25-27]. Targeted electroporation of the LV walls will prove to be useful to further explore the role of these transcription factors in the specification of postnatally generated OB neurons.

## Materials and methods

### Plasmid preparation

A pCX-GFP plasmid (kind gift of X Morin, ENS, Paris) coding for enhanced GFP under control of a chicken β-actin promoter and a cytomegalovirus enhancer was purified using the Qiagen EndoFree Plasmid Maxi Kit, according to the manufacturer's protocol. (Qiagen, Valencia, California, USA) Final plasmid preparation was made by re-suspending DNA in sterile phosphate-buffered saline at a concentration of 5 μg/μl. Fast green (Sigma-Aldrich, St. Louis, Missouri, USA) was added as contrasting agent at a final concentration of 1% to assess accuracy of the intraventricular injections.

### Postnatal electroporation

All experiments were performed in agreement with the Canton of Zurich veterinary office guidelines. All mice used in this study were of the CD1 strain (Swiss mice).

Electroporation of RGCs lining the LV was performed in postnatal-day-2 mice (P2), as described previously [8]. Briefly, following anesthesia by hypothermia, postnatal-day-2 pups were fixed on a custom made support plate placed in a stereotaxic rig. Injections were performed at the midpoint of a virtual line connecting the eye with the cranial landmark Lambda as visualized by a light source. Plasmid solution (1.5 μl) was injected at a depth of 2 mm from the skull surface using a Hamilton syringe equipped with a 34G needle.

The accuracy of the injection could be monitored by the filling of the injected ventricle by the dark solution. Only successfully injected mice were subjected to 5 electrical pulses (95 V, 50 ms, separated by 950 ms intervals) using the ECM 830 BTX electroporator (Harvard Apparatus, Holliston, Massachusetts, USA) and tweezer electrodes coated with conductive gel (Signa gel, Parker Laboratories, Fairfield, New Jersey, USA). Two different sizes of electrodes (5 and 10 mm diameter; BTX Tweezertrodes, Harvard Apparatus) were used. After electroporation, mice were placed in an incubator at 37°C until they fully recovered, before being returned to their mother.

### Tissue processing

Animals were sacrificed 1 or 21 dpe. Following fixation with 4% paraformaldehyde, brains were washed in TBS and embedded in 3% agarose in TBS (Tris-buffered saline). Sections were cut using a vibratome (Leica) at 50 μm for the 1-dpe forebrains and 30 μm for the OBs of adult mice, and collected as a series of three or eight, respectively. For the 1-dpe forebrains, one of every three sections was serially mounted on gelatine-coated slides and stored at 4°C until stained. All other sections were collected in 24-well plates in 0.1 M PB (phosphate buffer) for immunostaining. For long-term storage, OB sections were transferred to an antifreeze solution (25% glycerol, 25% ethylenglycol and 50% 0.1 M PB) and stored at -20°C until they were processed.

### Immunohistochemistry

For animals sacrificed at 1 dpe, the native GFP fluorescence was very intense, and no GFP signal enhancing immunostaining was required. For animals sacrificed at 21 dpe, immunostaining against GFP was performed and combined with other markers (as detailed in Table 1). All immunostainings were performed on free-floating sections. Sections were washed three times in 0.1 M PB for 5 minutes. Blocking and permeabilization were achieved by incubating the sections for 2 hours in 0.1 M PB, with 0.4% TitronX-100 (PB-Tx) and 5% heat inactivated normal horse serum in 0.1 M PB at room temperature. Afterwards, primary antibody incubations were performed in PB-Tx 3% horse serum at 4°C overnight (please refer to Table 1 for a complete list of antibodies and dilutions). After three washes, incubation with species-matched secondary antibodies was performed for 2 hours at 4°C in PB-Tx with 3% horse serum. GFP signal was amplified using a biotin-conjugated secondary antibody against the GFP primary antibody. The Streptavidin Alexa 488 complex, which binds to biotin, was then incubated together with the nuclear stainings. Sections were counterstained with 4',6-diamidino-2-phenylindole (DAPI, Sigma) and Topro3 (Invitrogen, Carlsbad, California, USA) and coverslipped with antifading mounting medium (Vectashield, Vector Labs, Burlingame, California, USA).

**Table 1:** Antibodies used in this study

Antibody	Species	Concentration	Source (catalogue number)
Primary			
αCB	Rabbit	1:5,000	Swant (D-28K)
αCR	Rabbit	1:2,000	Swant (7699/4)
αGFP	Chicken	1:1,000	Abcam (Ab13970)
αTH	Mouse	1:500	Millipore (MAB 318)
Secondary			
Biot. αchicken	Donkey	1:1,000	Jackson (703-065-155)
αMouse Alexa 555	Donkey	1:1,000	Invitrogen (A31570)
αRabbit Alexa 555	Donkey	1:1,000	Invitrogen (A31572)
αStreptavidin Alexa 488	Donkey	1:500	Invitrogen (532354)

Swant (Marly, Fribourg, Switzerland)

Abcam (Cambridge, County of Cambridgeshire, UK)

Millipore (Billerica, Massachusetts, USA)

Jackson (West Grove, Pennsylvania, USA)

Invitrogen (Carlsbad, California, USA)

### Analysis

To analyze the distribution of the electroporated cells in the LV and the newly generated neurons in the OB, mosaic pictures were acquired at 20 × (NA 0.5) on an epifluorescent Leica DM5500 microscope equipped with a motorized stage.

Co-localization of markers was analyzed on a Leica SPE - II confocal microscope equipped with a 40 × objective (NA 1.25)

All the analyses were conducted using Neurolucida and Neurolucida Explorer softwares (mbf Bioscience). For counting the number and analyzing the distribution of electroporated cells in defined walls of the LV, a minimum of five animals for each condition were used throughout. An average of nine sections was analyzed for each brain from the most rostral portion of the LV (that is, before the opening of the ventricles) up to the emergence of the dentate gyrus. To compare the distribution and phenotypes of newborn neurons originating from distinct LV walls, a minimum of five animals per condition were analyzed from two independent litters. Distribution of newborn neurons in the OB was analyzed by counting a minimum of 60 GFP+ cells per animal. Expression of neuronal subtype-specific markers (CR, CB, TH) was analyzed in a minimum of 35 PG and 40 granule cells per animal, selected randomly from an average of eight sections covering the entire OB rostro-caudal extent.

All data are expressed as mean ± standard error of the mean. *P*-values were determined using unpaired *t*-test (Prism 5, GraphPad Software, La Jolla, California). Each animal is equivalent to n = 1.

## Abbreviations

CB: calbindin; CR: calretinin; dpe: days post-electroporation; GCL: granule cell layer; GFP: green fluorescent protein; GL: glomerular layer; LV: lateral ventricle; NSC: neural stem cell; OB: olfactory bulb; PG: periglomerular; RGC: radial glial cell; SVZ: subventricular zone; TH: tyrosine hydroxylase.

## Competing interests

The authors declare that they have no competing interests.

## Authors' contributions

MEF, SC and OR carried out the experimental work. CB and HC contributed reagents and expertise for the electroporation experiments. OR conceived of the study. MEF, CB and OR wrote the manuscript. All authors read and approved the final manuscript.

## Supplementary Material

Additional file 1 **Figure S1: Illustration of the experimental procedure for the determination of the electroporated area and the number of electroporated cells**. One out of every three sections was serially mounted on gelatine-coated slides. **(A) **The brain and ventricle outlines were drawn using the Neurolucida software (mbf Bioscience). **(B,D) **Next, the position of GFP+ RGCs (B) was superimposed on the drawings, and a line defining the extension of electroporated area was added (D). **(C) **A three-dimensional representation of the drawing was generated using Neurolucida explorer, allowing accurate measurement of the electroporated area and a three-dimensional reconstruction. **(B',D') **Higher magnification views of the ventricles shown in (B,D), respectively. Click here for file

Additional file 2 **Video 1: Rotating three-dimensional reconstruction of a laterally electroporated brain**. The first rotations show the brain contours (grey) and the lateral ventricle contour (white). Then, the electroporated area is superimposed (green). Click here for file

Additional file 3 **Video 2: Rotating three-dimensional reconstruction of a dorsally electroporated brain**. The first rotations show the brain contours (grey) and the lateral ventricle contour (white). Then, the electroporated area is superimposed (green). Click here for file

Additional file 4 **Video 3: Rotating three-dimensional reconstruction of a septally electroporated brain**. The first rotations show the brain contours (grey) and the lateral ventricle contour (white). Then, the electroporated area is superimposed (green). Click here for file
