# Supplementary figures and images for: Targeted electroporation of defined lateral ventricular walls: a novel and rapid method to study fate specification during postnatal forebrain neurogenesis
Source: Neural Dev. 2011 Apr 5;6:13. doi: 10.1186/1749-8104-6-13 (PMC3098142; doi:10.1186/1749-8104-6-13)

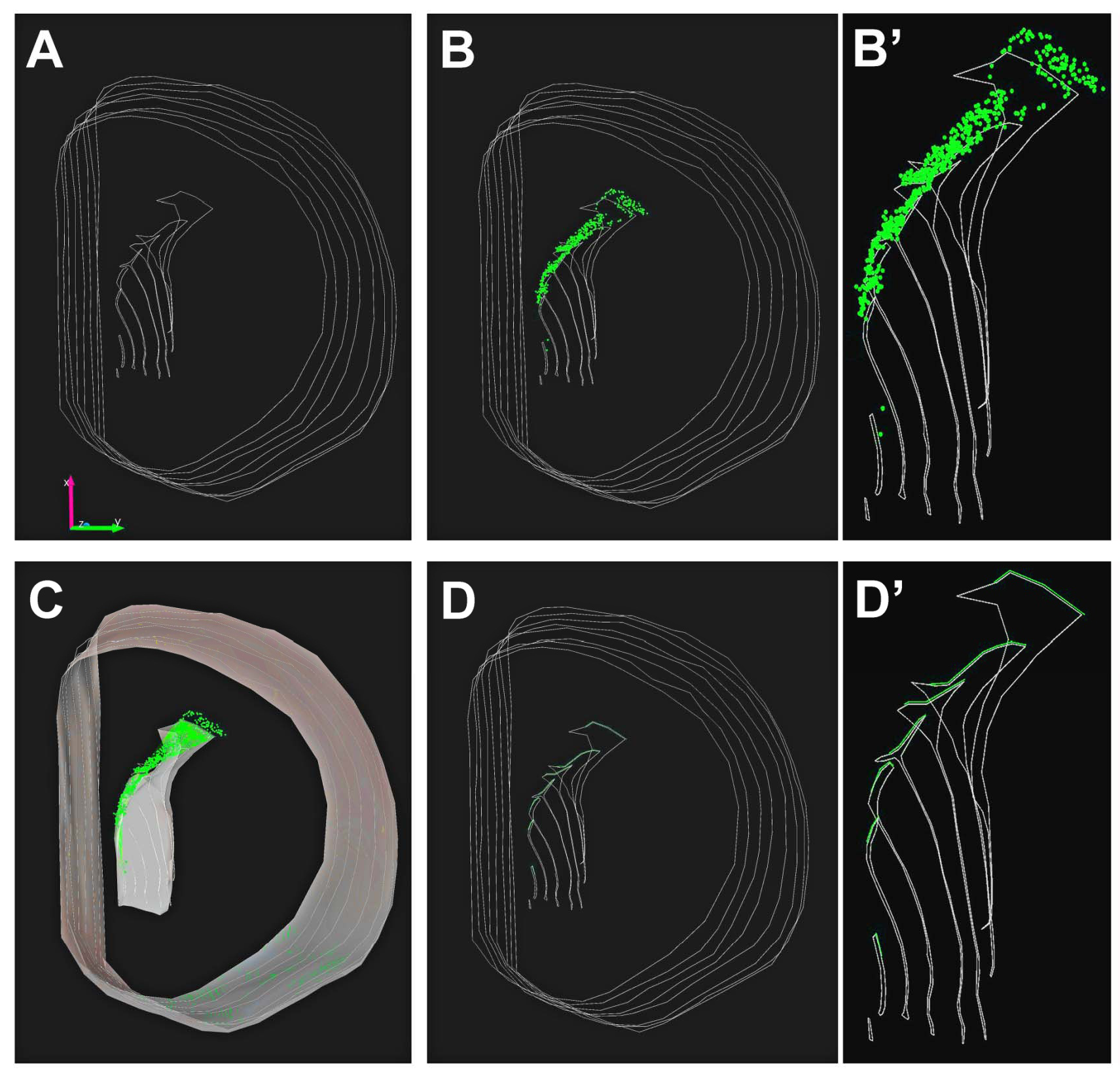

Supplement: Additional file 1 — Figure S1: Illustration of the experimental procedure for the determination of the electroporated area and the number of electroporated cells. One out of every three sections was serially mounted on gelatine-coated slides. (A) The brain and ventricle outlines were drawn using the Neurolucida software (mbf Bioscience). (B,D) Next, the position of GFP+ RGCs (B) was superimposed on the drawings, and a line defining the extension of electroporated area was added (D). (C) A three-dimensional representation of the drawing was generated using Neurolucida explorer, allowing accurate measurement of the electroporated area and a three-dimensional reconstruction. (B',D') Higher magnification views of the ventricles shown in (B,D), respectively. [file 1749-8104-6-13-S1.tiff]
